# Impact of interactions among metabolic syndrome components on the development of cardiovascular disease among Kazakhs in Xinjiang

**DOI:** 10.1371/journal.pone.0205703

**Published:** 2018-10-16

**Authors:** Wenwen Yang, Xiang Gao, Xianghui Zhang, Yunhua Hu, Heng Guo, Kui Wang, Yizhong Yan, Jia He, Jingyu Zhang, Jiaolong Ma, Lei Mao, Lati Mu, Jiaming Liu, Shugang Li, Yusong Ding, Mei Zhang, Rulin Ma, Shuxia Guo

**Affiliations:** 1 Department of Public Health, Shihezi University School of Medicine, Shihezi, Xinjiang, China; 2 Department of Nutritional Sciences, The Pennsylvania State University 109 Chandlee Lab, University Park, Pennsylvania, United States of America; 3 Departmentof Pathology and Key Laboratory of Xinjiang Endemic and Ethnic Diseases (Ministry of Education), Shihezi University School of Medicine, Shihezi, Xinjiang, China; The University of Texas at El Paso, UNITED STATES

## Abstract

**Background:**

Few prospective studies have explored the effect of interactions among metabolic syndrome (MS) components on the development of cardiovascular disease (CVD) in the Kazakh population in Xinjiang Province of China.

**Method:**

As of December 2016, 2,644 participants who completed a baseline survey over a period of 5 years or more were included in the study. The multiplicative interactions among MS components were evaluated by incorporation of the product terms into a logistic regression model. The additive interactions among MS components were evaluated by calculating the additive interaction index. Logistic regression was used to construct a predictive model, and CVD risk level was divided according to the risk probability of the population that did not eventually have CVD.

**Results:**

When we analyzed the independent risk of MS and its components on developing CVD, only blood pressure(BP) and waist circumference(WC) were associated with CVD. A linear association was found between the risk of CVD, BP/WC, and the number of other components (trend, P<0.001). The risk of developing CVD increased when BP and WC coexisted, or when combined BP/WC with MS (≥3 components except for BP and WC) was present; however, there were no significant interactions among MS components. After the CVD hazards were divided into four levels, it was showed that over 19.92% of the incidence probability was in the population under mediate-risk while over 35.24% of them was in the high-risk group, respectively.

**Conclusions:**

BP and WC were independent risk factors for CVD in the Kazakh population. The risk of CVD was greatly increased when BP and WC coexisted or when combined BP/WC with MS (≥3 components except for BP and WC) was present, but no significant interactions were found among MS components.

## 1. Introduction

Cardiovascular disease (CVD) accounts for about 40% of all deaths in China and creates a vast economic burden[1]. Numerous studies have shown that patients diagnosed with metabolic syndrome(MS)are more likely to have or develop CVD[2–7]. The above studies have shown that MS is a risk factor for CVD, but how it influences the risk of CVD is unclear. Therefore, there is a need to further explore the extent to which MS and its components impact the risk of CVD.

This study aimed to determine whether the risk of CVD caused by MS is greater than the sum of their components when multiple metabolic abnormalities coexist simultaneously, or whether MS predicts CVD independent of MS components. The above questions can be clarified by analyzing the interactions between MS components. Kang et al.[8]found that the risk of developing CVD was increased when blood pressure(BP)and other components coexisted, but no significant interaction was found between BP and other MS components. Liu et al.[9]found that BP and other MS components are only independent of each other for CVD, and there is no obvious interaction when these risk factors coexist. Both studies explored the interactions among MS components, but it is only the interaction between BP and other MS components. Therefore, the impact of the interactions between other MS components on CVD requires further study. Moreover, the subjects of the above two studies were Han Chinese. Due to the inherent differences in diet and genetic characteristics among ethnic groups, that conclusion may not be applicable to the Kazakhs.

Xinjiang, a northwest province of China, comprises 47 ethnic groups, of which the Kazakhs comprise the second largest group. Their special ethnicity, living environment, and genetic characteristics make the prevalence of MS and related diseases in the Kazakhs higher than that of other ethnic groups[10–13]. Because analyzing the interactions between MS components and CVD could identify whether the risk of CVD associated with MS is greater than the risk associated with its components, there are few similar well-designed prospective studies in China, particularly in Kazakh populations. Moreover, analyzing the impact of the interactions among MS components on CVD in the Kazakh population is important to the prevention and treatment of CVD in this population. Therefore, the present study, in the context of the multiple metabolic disorders in Kazakh populations in Xinjiang Province of China, explored these questions by analyzing the independent risk of each MS component for CVD and the interactions among MS components.

## 2. Method

### 2.1. Study population

In this study, data regarding the "Kazakh’s metabolic syndrome and its risk factors predicted risk of CVD in Xinjiang" were obtained. Briefly, the baseline study was conducted betweenApril2010 and December 2012 in the Xinyuan County, the Xinjiang Kazakhs Autonomous Region in China. A total of2,644 individuals participated in the baseline survey(2010–2012) and they were followed-up for more than 5 years. The second survey was conducted in 2016 and only 2,286 (out of 2,644) subjects were followed-up with a follow-up rate of 86.46%. A total of 281 individuals who had CVD (coronary heart disease [CHD], stroke, and hypertension)in the baseline survey were excluded from the study. Thus, as of December 2016, the remaining 2,005 subjects were included in the cohort analysis. Within the follow-up period, a total of 278 individuals developed CVD. Cardiovascular survival time was defined as the follow-up period from 2010 to the first occurrence of the event or end of follow-up, whichever came first.

### 2.2. Epidemiological survey and biochemical detection

All of the study subjects completed a demographic information survey questionnaire during face-to-face interviews. Detailed information about diet, drinking history, smoking history, disease details, and family history of disease were collected by trained investigators. According to standardized methods[14], height, weight, waist circumference(WC) and BP were measured by field workers. Fasting plasma glucose (FPG), triglyceride(TG) and high density lipoprotein cholesterol (HDL-C)levels based on overnight fasting blood samples collected during the survey were measured using a biochemical auto-analyzer (Olympus AU 2700; Olympus Diagnostics, Hamburg, Germany). All described methods were performed in accordance with the approved guidelines and regulations. All of the participants provided written informed consent prior to the start of the study. This study was approved by the Institutional Ethics Review Board (IERB) of the First Affiliated Hospital of Shihezi University School of Medicine (IERB No. SHZ2010LL01).

### 2.3. Diagnostic criteria for CVD

Subjects who met any of the following were diagnosed with CVD: first-ever occurrence of CHD, stroke, or hypertension during the follow-up period. The participants were hospitalized for CHD during the follow-up period; coronary intervention (cardiac catheterization or coronary bypass surgery) was performed; angina occurred (or nitroglycerin was initiated after cohort study); the first occurrence of myocardial infarction, and congestive heart failure were after the baseline investigation. Stroke was classified as an ischemic or hemorrhagic attack. Data on CVD events were obtained from patients’ hospital medical records and questionnaire responses. If the same kind of event occurred more than once, the first occurrence was considered the end event.

### 2.4. Definition of MS

MS was defined using the Third Report of the Adult Treatment Unit with a Modified US National Cholesterol Education Program (2005)[15]. MS is defined as meeting three or more of the following factors: (1) central obesity: WC≥90 and 80 cm for men and women, respectively; (2) elevated TG level (>150 mg/dL or 1.70 mmol/L), or have accepted the corresponding treatment; (3) reduced HDL-C(<40 mg/dL or 1.04 mmol/L in men,<50 mg/dL or 1.30 mmol/L in women)or have accepted the corresponding treatment; (4) elevated systolic BP (≥130 mmHg) or diastolic BP (≥85 mmHg)or have received the appropriate treatment for or was previously diagnosed with hypertension; (5) elevated FPG (≥100 mg/dL or 5.6 mmol/L) or have received the appropriate treatment for or was previously diagnosed with type 2 diabetes.

### 2.5. Confounding factors

Traditional risk factors used in the data analysis included sex, drinking history(current drinking, ever drinking, never drinking), family history of hypertension, family history of diabetes and family history of CVD.

### 2.6. Statistical analysis

EpiData3.02 software was used to establish a database, and we used a double entry data-in and logic error detection method. Data are presented as numbers(percentage) for categorical variables and as mean±standard deviation for continuous variables. Continuous variables between groups were compared by Student’s t-test, while categorical variables were analyzed using Chi-square test. A logistic regression model was used to evaluate the association between each MS component with the development of CVD. We also determined the association between BP/WC and other combinations of MS components with CVD. The trend test was performed using the Breslow and Day method [16].

The interactions between BP and WC and those between BP/WC and MS(≥3 components except for BP and WC) were evaluated. Therefore, subjects were divided on the basis of 2 separate factors and into 4 different subgroups to determine the risks of different combinations on the development of CVD and the interactions between them:

(1) without WC [WC (-)] and without BP [BP(-)];(2) with WC [WC (+)] and BP(-);(3) WC (-) and with BP [BP(+)];(4) WC (+) and BP(+).
(1) without MS [MS(-)] and BP(-);(2) with MS [MS(+)] and BP(-);(3) MS(-) and BP(+);(4)MS(+) and BP(+).
(1) MS(-) and WC (-);(2) MS(+) and WC (-);(3)MS(-) and WC (+);(4) MS(+) and WC (+).

The multiplicative interactions among MS components were evaluated by incorporation of the product terms into a logistic regression model. By calculating the parameter estimates and covariance matrix of the logistic regression model, Andersson’s [17] interactive calculation table was introduced to calculate the additive interaction index: the relative excess risk of interaction(RERI), attributable proportion of interaction(AP), synergy index(SI), and its confidence interval (CI), which was used to evaluate the additive interactions among the MS components. If there was no additive interaction, the CI of RERI and AP included 0, and the CI of SI contained 1.0. A logistic regression model was used to establish a CVD risk prediction model based on the interactions among MS components and divide the CVD hazard level. All statistical analyzes were performed using SPSS version 17.0 for Windows (SPSS Inc., Chicago, IL, USA). All statistical tests were two-sided, and a p-value< 0.05 was considered statistically significant.

## 3. Results

### 3.1. Baseline general characteristics of study subjects

The incidence of CVD in this study was 13.87%, while those of CVD in the MS and non-MS groups were 21.59% and 11.10%, respectively. Table 1 shows the baseline characteristics of the participants. There were significant differences between the MS and non-MS groups in most of the covariates(p<0.05) (Table 1).

**Table 1:** Baseline general characteristics of the study subjects.

Risk factor	MS	Non-MS	*P*
Sex, female/male	330/198	847/630	0.039
Age, years	46.33±12.04	38.71±11.83	<0.001
WC,cm	91.24±10.67	79.40±9.36	<0.001
SBP,mmHg	135.74±18.78	122.97±17.95	<0.001
DBP,mmHg	87.42±12.91	78.71±11.94	<0.001
HDL-C,mmol/L	1.21±0.38	1.41±0.54	<0.001
TG,mmol/L	1.79±1.56	1.06±0.96	<0.001
FPG,mmol/L	5.75±1.66	5.01±0.93	<0.001
Smoking rate,n(%)	195(36.93)	434(29.38)	0.001
Drinking rate,n(%)	80(15.15)	147(9.95)	0.001
Family history of hypertension,n(%)	206(39.02)	501(33.92)	0.035
Family history of diabetes,n(%)	9(1.70)	9(0.61)	0.022
Family history of CVD,n(%)	56(10.61)	113(7.65)	0.036
Incidence of CVD,n(%)	114(21.59)	164(11.10)	<0.001

WC, waist circumference; SBP, systolic blood pressure; DBP, diastolic blood pressure; HDL-C, high-density lipoprotein cholesterol; TG, triglyceride; FPG, fasting plasma glucose; MS, metabolic syndrome; CVD, cardiovascular diseases

### 3.2. Adjusted odds ratio for CVD and the MS with its components in the logistic regression model

Table 2 shows the odds ratio(OR)of each component of MS for predicting CVD in the logistic regression model. A univariate analysis showed that MS and its components were associated with CVD except for HDL-C(model 1). After the adjustment for sex, drinking history, family history of hypertension, family history of diabetes, and family history of CVD, MS, WC, BP, TG, and FPG were still significantly associated with CVD, but the adjusted odds ratio (aOR)for MS and its components in relation to CVD decreased(model 2). After the mutual adjustment for each MS component, BP and WC were still independently associated with CVD, whereas the other MS components were not associated with CVD(model 3)(Table 2).

**Table 2:** Adjusted OR for CVD and the MS components on a logistic regression model.

Risk factor	OR(95%CI)		
	Model 1	Model 2	Model 3
MS	2.21(1.69,2.87)	2.14(1.64,2.79)	-
WC	2.54(1.95,3.30)	2.43(1.86,3.18)	2.17(1.57,3.00)
BP	2.45(1.88,3.19)	2.56(1.95,3.34)	2.38(1.75,3.23)
HDL-C	1.18(0.92,1.53)	1.11(0.85,1.45)	1.06(0.76,1.47)
TG	1.58(1.18,2.12)	1.57(1.17,2.11)	1.41(0.98,2.02)
FPG	1.37(1.05,1.80)	1.37(1.05,1.80)	1.30(0.94,1.79)

OR, odds ratio; CI, confidence interval; BP, blood pressure. Other abbreviations are defined in Table 1. Model 1,univariate analysis; Model2,adjusted for sex, drinking history, family history of hypertension, family history of diabetes, family history of CVD; Model3,adjustedforModel2andtheotherfour MS components

### 3.3. Adjusted OR of BP/WC and other MS components associated with CVD

We analyzed the risk for CVD of BP combined with other MS components. In contrast with those participants without any MS components, the incidence of CVD increased from 5.56% to 37.50%. We found a linear association between BP and the number of other components for the aOR (from 2.56 to 9.29) of CVD (trend, P<0.001). Meanwhile, we analyzed the risk for CVD of WC combined with other MS components. We also found that a linear association between WC and the number of other components for the aOR (from 2.63 to 9.47) of CVD (trend, P<0.001)(Table 3).

**Table 3:** Adjusted OR of BP/WC and other MS components associated with CVD.

	CVD(n)	Incidence of CVD,(%)	Adjusted OR**(95%CI)	*P*
BP and other MS components associated with CVD				
0 component	17	5.56	Reference	
BP only*	24	12.83	2.56(1.33,4.92)	0.005
BP+1 other component*	61	18.94	3.73(2.12,6.57)	<0.001
BP+2 other components*	47	19.26	3.78(2.10,6.81)	<0.001
BP+3 other components*	30	25.21	5.45(2.86,10.38)	<0.001
BP+4 other components*	18	37.50	9.29(4.31,20.04)	<0.001
P for trend	<0.001			
1–4 components without BP*	81	10.40	1.77(1.03,3.06)	0.04
WC and other MS components associated with CVD				
0 component	17	5.56	Reference	
WC only*	19	14.18	2.63(1.32,5.27)	0.006
WC+1 other component*	53	17.85	3.41(1.91,6.08)	<0.001
WC+2 other components*	54	20.15	3.97(2.22,7.09)	<0.001
WC+3 other components*	33	24.44	5.32(2.83,9.98)	<0.001
WC+4 other components*	18	37.50	9.47(4.40,20.40)	<0.001
P for trend	<0.001			
1–4 components without WC*	84	10.28	1.89(1.10,3.25)	0.021

*Compared with 0 components of MS;

**adjusted for sex, drinking history, family history of hypertension, family history of diabetes, and family history of CVD on logistic regression. Abbreviations are defined in Tables 1 and 2.

### 3.4. Interactions analysis between BP and WC and BP/WC and MS (≥3 components except for BP and WC) associated with CVD

We evaluated the effect ofthe interaction between BP and WC on CVD development. Next, the 2 separate factors, whichcomprised 4 different subgroups, were analyzed in pairs in the logistic regression model. After the adjustment for sex, drinking history, family history of hypertension, family history of diabetes, family history of CVD,the aOR between CVD and BP was 2.40(95% confidence interval [CI], 1.58–3.65) and between CVD and WC was 2.25(95%CI, 1.47–3.45). When BP and WC coexisted, the aOR was 4.82(95%CI, 3.32–6.99)(Table 4). Also, the indexof addictive interaction, RERIwas 1.16 (95%CI, -0.20–2.52), AP was 0.24 (95%CI, -0.02–0.50) and SI was 1.44 (95%CI, 0.91–2.28), which indicated no addictive interaction between the 2 risk factors(Table 5). The interaction analysis between BP/WC and MS (≥3 components except for BP and WC) on CVD development was performed. We found no additive interaction between BP/WC and MS (≥3 components except for BP and WC)(Table 5).

**Table 4:** Multiplying interactions analysis among BP and WC and BP/WC and MS (≥3 components except for BP and WC) associated with CVD.

Interpretation	CVD(n)	Incidence of CVD,(%)	Adjusted OR*(95%CI)	*P*
Interaction between BP and WC				
BP(-)and WC(-)	43	6.20	Reference	
BP(+)and WC(-)	58	13.49	2.40(1.58,3.65)	<0.001
BP(-)and WC(+)	55	14.03	2.25(1.47,3.45)	<0.001
BP(+)and WC(+)	122	24.90	4.82(3.32,6.99)	<0.001
Interaction between BP and MS				
BP(-)and MS(-)	90	8.61	Reference	
BP(+)and MS(-)	159	18.47	2.51(1.90,3.32)	<0.001
BP(-)and MS(+)	8	20.00	2.54(1.13,5.70)	0.024
BP(+)and MS(+)	21	35.59	5.77(3.22,10.32)	<0.001
Interaction between BP and MS				
WC(-)and MS(-)	96	8.74	Reference	
WC(+)and MS(-)	153	18.96	2.34(1.77,3.09)	<0.001
WC(-)and MS(+)	5	20.83	2.54(0.92,6.98)	0.072
WC(+)and MS(+)	24	32.00	4.68(2.74,7.97)	<0.001

*Adjusted for sex, drinking history, family history of hypertension, family history of diabetes, family history of CVD. MS(-),≤2 components of MSexcept BP and WC; MS(+),≥3 components of MS except BP and WC; BP(-),without BP;BP(+),with BP only; WC(-),without WC;WC(+),with WC only. Other abbreviations are defined in Tables 1 and 2.

**Table 5:** Additive interactions among MS components.

	Relative excess risk		Attributable proportion		Synergy index	
	Point estimation	95%CI	Point estimation	95%CI	Point estimation	95%CI
BP and WC	1.16	(-0.20,2.52)	0.24	(-0.02,0.50)	1.44	(0.91,2.28)
BP and MS*	1.72	(-1.99,5.43)	0.30	(-0.22,0.82)	1.56	(0.61,3.99)
WC and MS*	0.80	(-2.62,4.22)	0.17	(-0.51,0.85)	1.28	(0.43,3.78)

*≥3 components of MS except BP and WC; Abbreviations are defined in Tables 1 and 2.

### 3.5. Prediction probability of CVD and division of risk level

The calculation of prediction probability according to the logistic regression model is shown in Table 6. The risk level was divided based on a statistical description of risk probability of the population that did not eventually have CVD. Due to a skewed distribution of the predicted morbidity probability of healthy people,it is necessary to normalize the predicted probability (taking the natural logarithm) when dividing the risk level: $<\bar{X}-\text{S}$ are low risk, $\bar{X}-\text{S}$ to $\bar{X}+\text{S}$ are general risk, $\bar{X}+\text{S}$ to $\bar{X}+2\text{S}$ are moderate risk, and $>\bar{X}+2\text{S}$ are high risk. Next, the corresponding threshold value is inversed to the natural logarithm to obtain the corresponding relationship between the predicted probability and the risk level as shown in Table 7.

**Table 6:** Multi-factors logistic regression results.

Risk factor	β	OR(95%CI)	*P*
Constant	-3.41		<0.001
TG	0.26	1.29(0.93,1.79)	0.121
HDL-C	-0.03	0.97(0.73,1.29)	0.837
FPG	0.18	1.20(0.91,1.58)	0.207
BP(-)and WC(-)		1.00	-
BP(+)and WC(-)	0.87	2.38(1.56,3.63)	<0.001
BP(-)and WC(+)	0.76	2.14(1.39,3.29)	0.001
BP(+)and WC(+)	1.51	4.52(3.10,6.59)	<0.001
Sex	0.39	1.47(1.07,2.01)	0.016
Drinking history	0.19	1.21(0.78,1.88)	0.396
Family history of CVD	0.39	1.48(0.97,2.24)	0.067
Family history of diabetes	-0.38	0.68(0.15,3.11)	0.620
Family history of hypertension	-0.08	0.93(0.70,1.22)	0.578

Abbreviations are defined in Tables 1 and 2.

**Table 7:** Prediction probability of CVD and division of risk level.

	Low risk	General risk	Moderate risk	High risk
Probability of prediction(%)	<(0–6.36)	<(6.36–19.92)	<(19.92–35.24)	35.24–100.00
Corresponding risk level	1	2	3	4
Number and proportion of each risk level	336(19.46)	1052(60.91)	330(19.11)	9(0.52)

## 3. Discussion

Numerous previous studies found that MS is a risk factor for CVD[5, 6, 18–21]. Our investigation also showed that MS is associated with CVD and this association persisted even after the adjustment for sex, drinking history, family history of hypertension, family history of diabetes, and family history of CVD. Recent studies investigated the independent association of MS and its components for predicting CVD[20, 22]. A study of a Swedish population bySundstrom et al.[23], suggested that the association between MS and CVD was not significantwhen general risk factors and MS components were adjusted, a finding that is consistent with the results of our study. These studies suggested that MS does not provide more information about predicting CVD than its individual components.

Which component of MS was associated with CVD to a larger degree? A cohort study of 11 provinces in China showed that BP is the most important risk factor for CVD in the Chinese population[24]. Chen et al.[7] also found that BP was the hard core of MS contributing to an increased risk of CVD. In the AsiaPacific region, up to 66% of some CVD subtypes can be attributed to hypertension[25]. The present study found that allMS componentsdo not carry equal risksfor CVD development. After the adjustment for the general risk factors of CVD, the strength of the connection between BP and CVD is close to that of MS and CVD (model 2). However, when the general risk factors and MS components were adjusted, the relationship between MS and CVD was not significant and only BPwas independently associated with CVD(model 3). In addition, in recent years, studies in other countries reached the same conclusion[26, 27]. Lawlor[28]reported on aheart and health study of women in the United Kingdom thatafter the adjustment for general risk factors and MS components, only BP(relative risk = 1.16: 95% CI, 1.00–1.35)was associated with CHD, and the association between MS and CVD was not significant. These phenomena further suggested that the ability of MS to predict the risk of CVD is not independent of its individual components. It is relatively consistent that BP is an independent risk factor for the development of CVD. The present study also found that WC was independently associated with CVD after the adjustment for general risk factors and MS components. The probable reason for this is the high prevalence of central obesity in the Kazakh population[12].

We further analyzed the risk for CVD of BP/WC combined with other MS components. We found a linear association between BP and the number of other components for the aOR (from 2.56 to 9.29) of CVD and between WC and the number of other components for the aOR (from 2.63 to 9.47) of CVD. This finding implied a cumulative effect of increasing the risk of CVD, which indicated that when the individual’sBP/WC indexwas abnormal, the more other MS components were present, the greater the risk of CVD.

Our investigation found that BP and WC were independent risk factors for CVD development in the Kazakh population, which required further consideration of whether BP and WC, BP/WC, and MS (≥3 components except BP and WC)interactfor cardiovascular risk. Therefore, we analyzed the interaction between BP and WC or between BP/WC and MS (≥3 components except BP and WC) and explored whether there is an additional risk of CVD when these factors coexisted. The present study found thatwhenBP and WC coexisted, the aOR was 4.82(95% CI, 3.32–6.99), which indicated thatthe accumulation of BP and WC also increases the risk of CVD. However, after examining the additive interaction, three additive interaction indexes showed no additive interactionbetween BP and WC. The present study also foundno observable biological interactionbetween WC and MS (≥3 components except for BP and WC). Simultaneously, we also found no observable biological interaction betweenBP and MS (≥3 components except for BP and WC). This result was consistent with the results obtained by Kang et al.[20], who reportedthe impact of BP and other MS components on the development of CVD. Liu et al.[9] reported the association between MS components and CVD, which indicatedno observable biological interaction between BP and other MScomponents. The above phenomenon suggestedthat when multiple metabolic abnormalities occur at the same time, the risk of CVD caused by MS is not greater than the sum of their components, that is, risk factors such as BP, WC, and other MS components only play independent roles in CVD, indicating that the presence of MS does not provide a clinician with more or better information. The interactionsamongBP, WC, and MS(≥3 components except for BP and WC)for predicting CVD are not significant. Itmay be because the MS components have a common physiological basis, such as insulin resistance,[29] inflammation,[30] or central obesity,[31] or they are closely related to each other.

The present study divided CVDhazards into four levels to identify CVD high-risk groups and provide a scientific basis for better CVD prevention and treatment. Our investigation found that 0–6.36% of the incidence probability was in the low-riskpopulation, 6.36–19.92% in the general-riskpopulation, 19.92–35.24% in the moderate-riskpopulation, and 35.24~100.00% in the high-riskpopulation. Moreover, for participantsin the low- or general-riskpopulations, primary prevention should be performed. For moderate- and high-riskparticipants, secondary prevention should be performed. The classification of CVD hazard level provides an easy assessment tool forhealth education and health promotion in the Kazakh population. The early identification of populationsat high risk of developing CVD plays an important role in decreasing the incidence of CVD and improving quality of life.The risk classification of CVD can also provide an important reference for the future development of an effective and feasible intervention measure for chronic diseases such as CVD in Kazakhs in Xinjiang.

The strength of the present study is the fact that this is the first Kazakh population-based study to explore the effect of interactions among MS components on the development of CVD. The study found that BP and WC were independent risk factors for CVD in the Kazakh population and identified a linear association between the risk of CVD, BP/WC, and the number of other components. The study found that the risk of developing CVD increased when BP and WC coexisted or BP/WCwas combined with MS (≥3 components except for BP and WC), but there were no significant interactions among MS components in the Kazakh population. The study found that over 19.92% of the incidence probability was in the population under mediate-riskwhile over 35.24% of them was in the high risk group, respectively. However, due to site-specific limitations, the CVD outcomes analyzed here failed to include hospitalization or outpatient visits due to a transient myocardial ischemia that prevented symptom remission, which may have led to the underestimation of the incidence of CVD.

## 5. Conclusions

BP and WC were independent risk factors for CVD in this Kazakh population. The risk of CVD was greatly increased when BP and WC coexisted orBP/WCwascombined with MS (≥3 components except for BP and WC), but no significant interactions were found among MS components.

## Supporting information

S1 File (XLS)

S2 File (SAV)
